# Supplementary material for: Identifying correlates of Guinea worm (Dracunculus medinensis) infection in domestic dog populations
Source: PLoS Negl Trop Dis. 2020 Sep 14;14(9):e0008620. doi: 10.1371/journal.pntd.0008620 (PMC7515199; doi:10.1371/journal.pntd.0008620)
Supplement: S1 Table — A list of the source, resolution, and means (+/- SE), where appropriate, for all variables included in the boosted regression tree models. Climatic, surface water, and floodwater summaries were calculated from monthly minimum, maximum, mean, and total values using the protocol of [28]. Climatic variables in the main text use WorldClim 2.0 data while additional present climate analyses were trained on present climate data with a coarser spatial grain. Variables included after co-linearity reduction are indicated in bold. (PDF) [file pntd.0008620.s001.pdf]

Variable	Source	Resolution	All Villages N = 2125	Northern Villages N = 1605	Southern Villages N = 520
**Demographic**					
**Dog population**	Guinea worm survey	Village	27.28 dogs (1.02)	17.65 dogs (0.77)	56.06 dogs (3.07)
**Fishing Village identity (Fishing)**	Guinea worm survey	Village	30.6%	33.5%	21.8%
**Human population size (HumanPopulation)**	Guinea worm survey	Village	484.55 humans (22.52)	422.75 humans (23.73)	675.81 humans (54.98)
**Modeled Gridded Human Population Size (RemotePop)**	NASA SEDAC	1 km	23.79 humans (0.33)	24.41 humans (0.41)	21.87 humans (0.43)
Number of households(NoHouseholds)	Guinea worm survey	Village	105.40 households (4.66)	90.24 (4.25)	152.29 households (13.59)
**Geographic**					
Latitude	Guinea worm survey	Village			
Longitude	Guinea worm survey	Village			
**Northern or Southern Village (NS)**	Guinea worm survey	Village			
**Landcover type (LandCover)**	GLOBCOVER 2009	~300m			
Mean Elevation (MeanElev)	NASA SRTM	~30m aggregated to 1km village buffer	343.66 m (0.81)	333.30 m (0.92)	375.65 m (0.59)
**Standard Deviation in Elevation (ElevSD)**	NASA SRTM	~30m aggregated to 1km village buffer	2.99 m (0.06)	2.66 m (0.05)	4.00 m (0.20)
**Permanent water area (WaterArea)**	NASA SEDAC	1km	0.024 km^2 (0.001)	0.028 km^2 (0.002)	0.008 km^2 (0.002)
**Distance to nearest permanent water (RiverDist)**	Hydrosheds database	Vector	1488.45 m (24.83)	1528.29 m (30.60)	1365.48 m (46.80)
**Mean annual surface water (SurfaceWaterMean)**	NRT Global MODIS Flood Mapping	~250m aggregated to 1km village buffer	133.49 m^2 (0.27)	136.98 m^2 (0.29)	122.70 m^2 (0.37)
**Maximum surface water of the wettest month (SurfaceWaterMax)**	NRT Global MODIS Flood Mapping	~250m aggregated to 1km village buffer	234.37 m^2 (0.38)	235.15 m^2 (0.48)	231.97 m^2 (0.43)
Minimum surface water of the driest month (SurfaceWaterMin)	NRT Global MODIS Flood Mapping	~250m aggregated to 1km village buffer	0 m^2 (0)	0 m^2 (0)	0 m^2 (0)
**Maximum surface water of the wettest quarter (SurfaceWaterQMax)**	NRT Global MODIS Flood Mapping	~250m aggregated to 1km village buffer	220 m^2 (0.30)	221.01 m^2 (0.37)	220.43 m^2 (0.41)
Minimum surface water of the driest quarter (SurfaceWaterQMin)	NRT Global MODIS Flood Mapping	~250m aggregated to 1km village buffer	0 m^2 (0)	0 m^2 (0)	0 m^2 (0)
**Climatic**					
Annual mean temperature (Bioclim1)	WorldClim 2.0/MERRA-2	~1km/~55km	27.90 °C (0.01)	28.07 °C (0.01)	27.39 °C (0.02)
Mean diurnal temperature range (Bioclim2)	WorldClim 2.0	~1km	14.14 °C (0.02)	14.43 °C (0.02)	12.22 °C (0.02)
Isothermality (Bioclim3)	WorldClim 2.0	~1km	67.84 (0.05)	66.87 (0.04)	70.83 (0.06)
Temperature seasonality (Bioclim4)	WorldClim 2.0	~1km	232.70 °C (0.62)	244.69 °C (0.50)	195.70 °C (0.64)
Maximum temperature of the wettest month (Bioclim5)	WorldClim 2.0	~1km	37.61 °C (0.03)	38.16 °C (0.02)	35.94 °C (0.03)
Minimum temperature of the coldest month (Bioclim6)	WorldClim 2.0	~1km	16.73 °C (0.01)	16.56 °C (0.01)	17.26 °C (0.01)
Temperature annual range (Bioclim7)	WorldClim 2.0/Merra-2	~1km/~55km	20.88 °C (0.03)	21.60 °C (0.03)	18.68 °C (0.03)
Mean temperatue of the wettest quarter (Bioclim8)	WorldClim 2.0/MERRA-2 and ARC2	~1km/~55km and ~11km	26.48 °C (0.01)	26.71 °C (0.01)	25.75 °C (0.01)
**Mean temperature of the driest quarter (Bioclim9)**	WorldClim 2.0/MERRA-2 and ARC2	~1km/~55km and ~11km	26.47 °C (0.01)	26.43 °C (0.01)	26.56 °C (0.02)
Mean temperature of the warmest quarter (Bioclim10)	WorldClim 2.0/MERRA-2 and ARC2	~1km/~55km and ~11km	31.25 °C (0.02)	31.56 °C (0.01)	30.31 °C (0.03)
**Mean temperature of the coldest quarter (Bioclim11)**	WorldClim 2.0/MERRA-2 and ARC2	~1km/~55km and ~11km	25.71 °C (0.01)	25.71 °C (0.01)	25.72 °C (0.01)
**Annual precipitation (Bioclim12)**	WorldClim 2.0/ARC2	~1km/~11km	786.60 mm (3.50)	716.40 mm (2.93)	1003.13 mm (1.69)
Precipitation of the wettest month (Bioclim13)	WorldClim 2.0/ARC2	~1km/~11km	227.99 mm (0.52)	222.21 mm (0.61)	245.85 mm (0.40)
Precipitation of the driest month (Bioclim14)	WorldClim 2.0/ARC2	~1km/~11km	0 mm (0)	0 mm (0)	0 mm (0)
Precipitation seasonality (Bioclim15)	WorldClim 2.0/ARC2	~1km/~11km	123.20 mm (0.21)	127.55 mm (0.17)	109.77 mm (0.10)
Precipitation of the wettest quarter (Bioclim16)	WorldClim 2.0/ARC2	~1km/~11km	556.14 mm (1.76)	523.46 mm (1.612)	656.94 mm (1.03)
Precipitation of the driest quarter (Bioclim17)	WorldClim 2.0/ARC2	~1km/~11km	0.39 mm (0.01)	0.11 mm (0.01)	1.24 mm (0.02)
Precipitation of the warmest quarter (Bioclim18)	WorldClim 2.0/MERRA-2 and ARC2	~1km/~55km and ~11km	93.74 mm (0.70)	78.97 mm (0.53)	139.30 mm (0.38)
Precipitation of the coldest quarter (Bioclim19)	WorldClim 2.0/MERRA-2 and ARC2	~1km/~55km and ~11km	146.00 mm (5.84)	18.14 mm (2.47)	540.40 mm (10.73)
**Surveillance**					
**Mean number of resident healthcare workers (NoASVTot)**	Guinea worm survey	Village	9.07 healthcare workers (0.32)	8.89 healthcare workers (0.30)	9.63 healthcare workers (0.91)
**Total number of healthcare supervisor visits to a village 2013-2017 (ASVVisits)**	Guinea worm survey	Village	94.74 visits (3.06)	93.50 visits (3.58)	98.57 visits (5.90)
