# Supplementary material for: Identifying correlates of Guinea worm (Dracunculus medinensis) infection in domestic dog populations
Source: PLoS Negl Trop Dis. 2020 Sep 14;14(9):e0008620. doi: 10.1371/journal.pntd.0008620 (PMC7515199; doi:10.1371/journal.pntd.0008620)
Supplement: S2 Table — This table reports the identity of covariates in each of two identified co-linear clusters. The central variable in each cluster, with the highest mean correlation with all other variables in the cluster, used to represent the cluster in analyses, appears in bold;the Pearson correlation between each covariate and that central variable is also reported. (PDF) [file pntd.0008620.s002.pdf]

Variable	Pearson Correlation with Central Variable
Cluster 1: **Annual Precipitation**	
Latitude	-0.970
Longitude	0.756
Mean Elevation	0.788
Annual mean temperature (BIO1)	-0.761
Mean diurnal temperature range (BIO2)	-0.839
Isothermality (BIO3)	0.727
Temperature seasonality (BIO4)	-0.894
Maximum temperature of the wettest month (BIO5)	-0.933
Minimum temperature of the coldest month (BIO6)	0.623
Temperature annual range (BIO7)	-0.937
Mean Temperature of wettest quarter (BIO8)	0.808
Mean Temperature of warmest quarter (BIO10)	0.536
Annual precipitation (BIO12)	1.000
Precipitation of the wettest month (BIO13)	0.851
Precipitation seasonality (BIO15)	-0.972
Precipitation of the wettest quarter (BIO16)	0.990
Precipitation of the driest quarter (BIO17)	0.772
Precipitation of the warmest quarter (BIO18)	0.843
Precipitation of coldest quarter (BIO19)	0.735
Cluster 2: **Human Population**	
Human Population	1.000
Number of Households	0.898
