# Supplementary material for: Identifying correlates of Guinea worm (Dracunculus medinensis) infection in domestic dog populations
Source: PLoS Negl Trop Dis. 2020 Sep 14;14(9):e0008620. doi: 10.1371/journal.pntd.0008620 (PMC7515199; doi:10.1371/journal.pntd.0008620)
Supplement: S3 Table — This table reports the interaction strength of top ranked pair-wise interactions in the boosted regression tree model for infection presence at the village scale. (PDF) [file pntd.0008620.s003.pdf]

Variable 1	Variable 2	Interaction Size
RemotePop	Fishing	10.43
Bioclim11	Fishing	5.73
ASVVisits	ElevSD	5.69
ASVVisits	DogPopulation	3.58
Bioclim12	DogPopulation	3.47
Bioclim9	DogPopulation	3.25
DogPopulation	Fishing	2.92
SurfaceWaterMean	Bioclim12	2.71
Bioclim12	Fishing	2.65
RemotePop	DogPopulation	2.12
RiverDist	Fishing	1.82
Bioclim11	Bioclim9	1.65
Bioclim9	LandCover	1.32
ASVVisits	Fishing	1.31
