# Supplementary material for: Identifying correlates of Guinea worm (Dracunculus medinensis) infection in domestic dog populations
Source: PLoS Negl Trop Dis. 2020 Sep 14;14(9):e0008620. doi: 10.1371/journal.pntd.0008620 (PMC7515199; doi:10.1371/journal.pntd.0008620)
Supplement: S4 Table — This table reports the interaction strength of top ranked pair-wise interactions in the boosted regression tree model for hotspot identity. (PDF) [file pntd.0008620.s004.pdf]

Variable 1	Variable 2	Interaction Size
Bioclim12	Bioclim11	1100.83
Bioclim11	Bioclim9	164.91
Bioclim12	Bioclim9	134.10
ASVVisits	Bioclim12	109.05
ASVVisits	Bioclim9	65.54
Bioclim11	ElevSD	65.20
Bioclim9	ElevSD	62.42
Bioclim12	RemotePop	56.08
SurfaceWaterMean	Bioclim12	51.52
SurfaceWaterMean	Bioclim11	50.34
SurfaceWaterQMax	Fishing	39.55
Bioclim9	LandCover	34.97
Bioclim12	ElevSD	25.75
Bioclim9	Fishing	16.53
