# Supplementary material for: Identifying correlates of Guinea worm (Dracunculus medinensis) infection in domestic dog populations
Source: PLoS Negl Trop Dis. 2020 Sep 14;14(9):e0008620. doi: 10.1371/journal.pntd.0008620 (PMC7515199; doi:10.1371/journal.pntd.0008620)
Supplement: S5 Table — This table reports the identity of covariates in each of two identified co-linear clusters. The central variable in each cluster, with the highest mean correlation with all other variables in the cluster, used to represent the cluster in analyses, appears in bold; the Pearson correlation between each covariate and that central variable is also reported. (PDF) [file pntd.0008620.s005.pdf]

Variable	Pearson Correlation with Central Variable
Cluster 1: **Maximum Temperature of Warmest Month**	
Latitude	0.771
Longitude	0.722
Mean Elevation	0.761
Annual mean temperature (BIO1)	0.674
Mean diurnal temperature range (BIO2)	0.445
Maximum Temperature of the Warmest Month (BIO5) 1.00	
Minimum temperature of the coldest month (BIO6)	0.143
Temperature annual range (BIO7)	0.670
Mean Temperature of wettest quarter (BIO8)	0.791
Mean Temperature of the Driest Quarter (BIO9)	0.534
Mean Temperature of warmest quarter (BIO10)	0.581
Mean Temperature of the Coldest Quarter (BIO11)	0.297
Annual precipitation (BIO12)	0.784
Precipitation of the wettest month (BIO13)	0.464
Precipitation seasonality (BIO15)	0.761
Precipitation of the wettest quarter (BIO16)	0.584
Precipitation of coldest quarter (BIO19)	0.7685
Cluster 2: **Human Population**	
Human Population	1.000
Number of Households	0.898
