# Supplementary figures and images for: Identifying correlates of Guinea worm (Dracunculus medinensis) infection in domestic dog populations
Source: PLoS Negl Trop Dis. 2020 Sep 14;14(9):e0008620. doi: 10.1371/journal.pntd.0008620 (PMC7515199; doi:10.1371/journal.pntd.0008620)

A

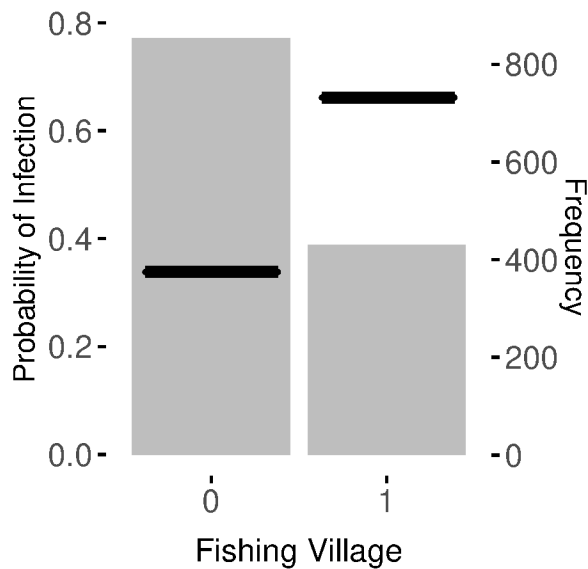

B

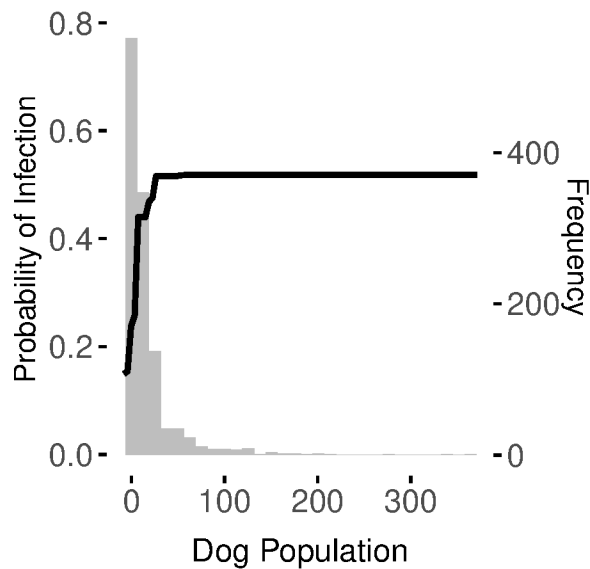

C

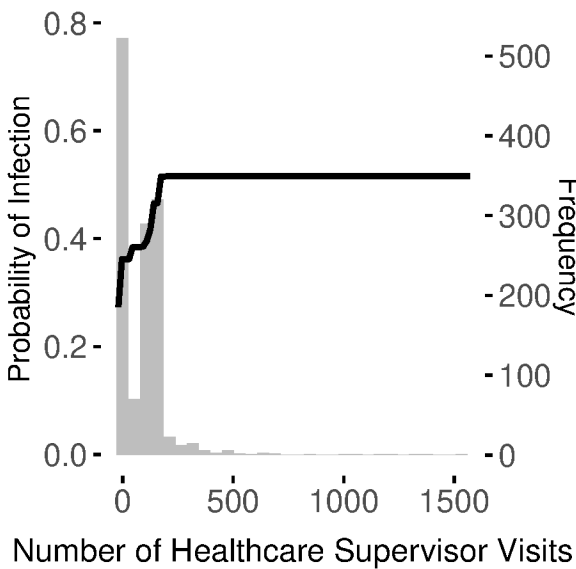

D

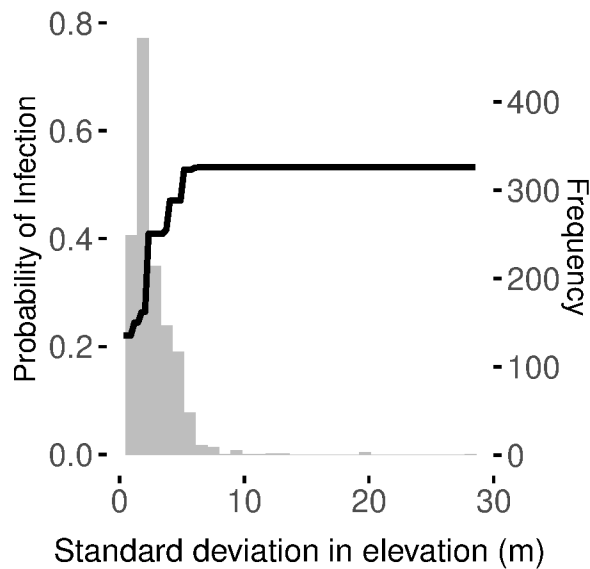

Supplement: S1 Fig — This figure depicts partial dependence plots showing the effect of (a) identity as a fishing village, (b) dog population, (c) number of healthcare supervisor visits, and (d) standard deviation in elevation on probability of parasite presence in northern villages. Histograms represent the distribution of values for these covariates amongst all training villages. (PDF) [file pntd.0008620.s006.pdf]

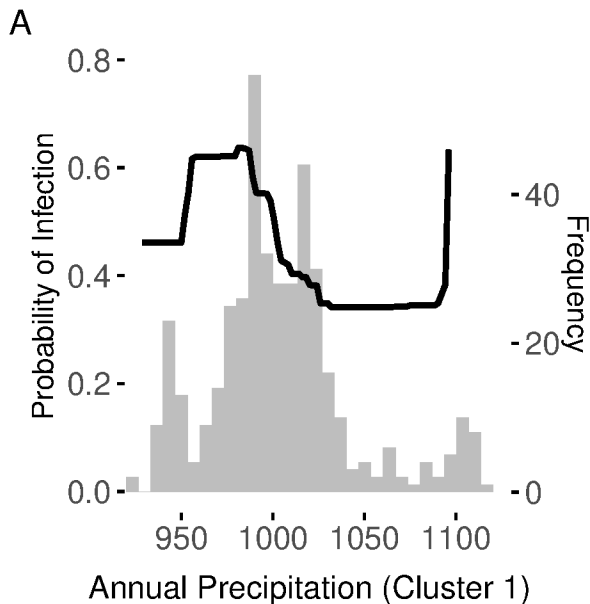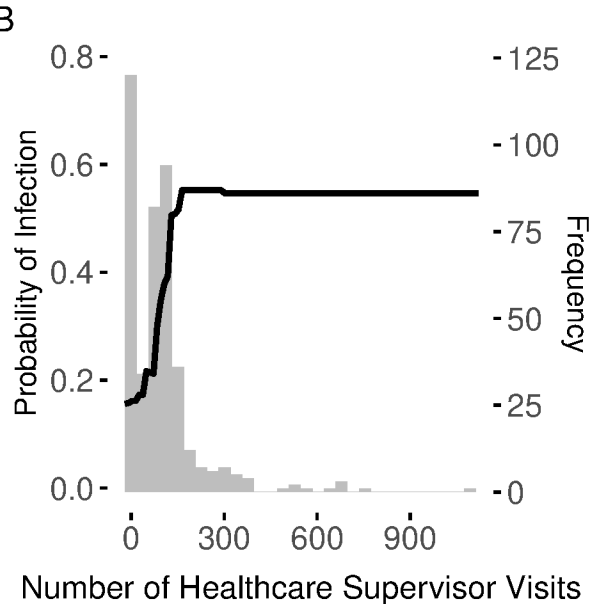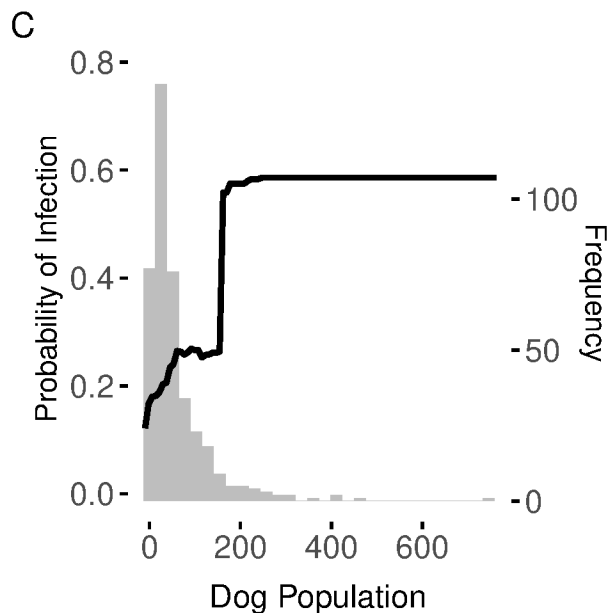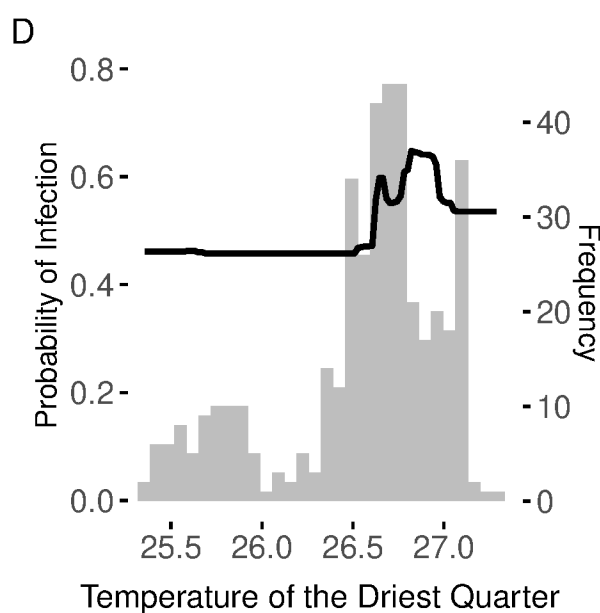

Supplement: S2 Fig — Partial dependence plots showing the effect of (a) Cluster 1 (represented by annual precipitation [Bioclim12]), (b) number of healthcare supervisor visits, (c) dog population, and (d) temperature of the driest quarter on probability of parasite presence in southern villages. Histograms represent the distribution of values for these covariates amongst all training villages. (PDF) [file pntd.0008620.s007.pdf]

A

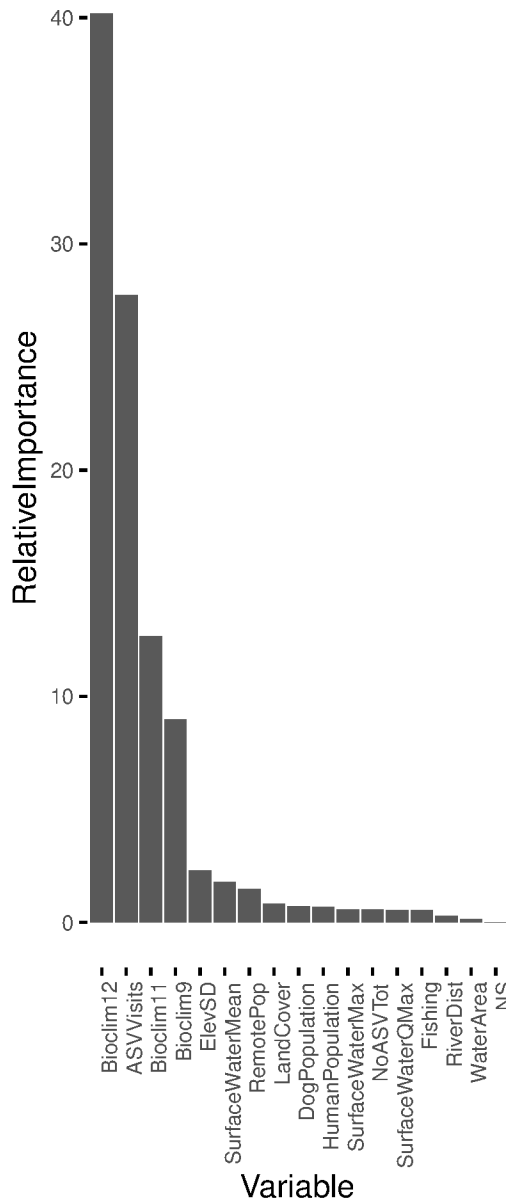

B

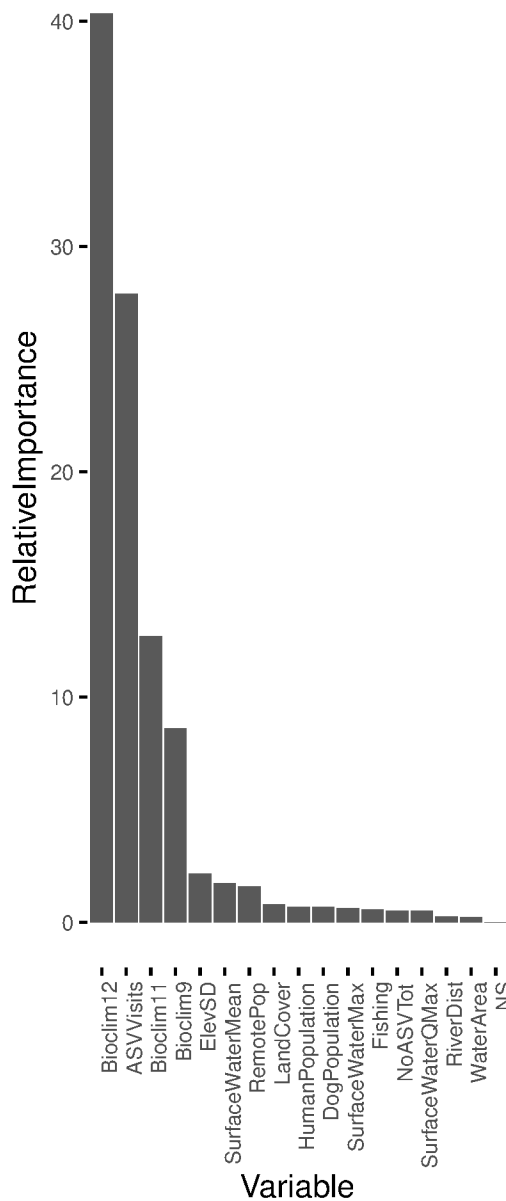

Supplement: S3 Fig — This figure depicts relative importance of covariates in predicting hotspot identity in (a) northern and (b) southern villages. (PDF) [file pntd.0008620.s008.pdf]

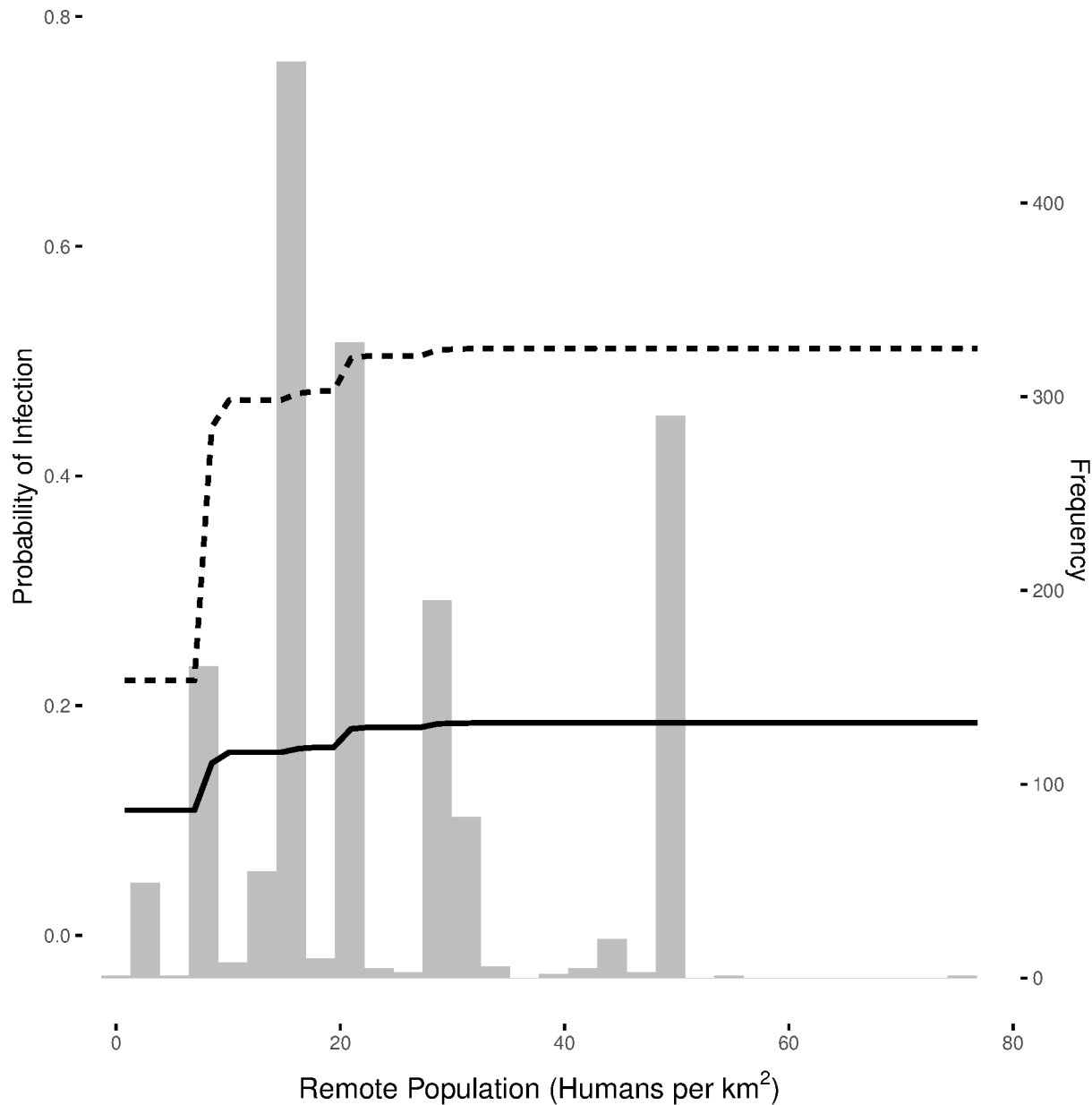

Supplement: S4 Fig — This figure depicts the strongest pair-wise interaction in the boosted regression tree model for parasite presence, between remotely sensed human population (x-axis) and fishing village identity. The dotted line represents villages designated as fishing villages and the solid line those not designated as fishing villages. (PDF) [file pntd.0008620.s009.pdf]

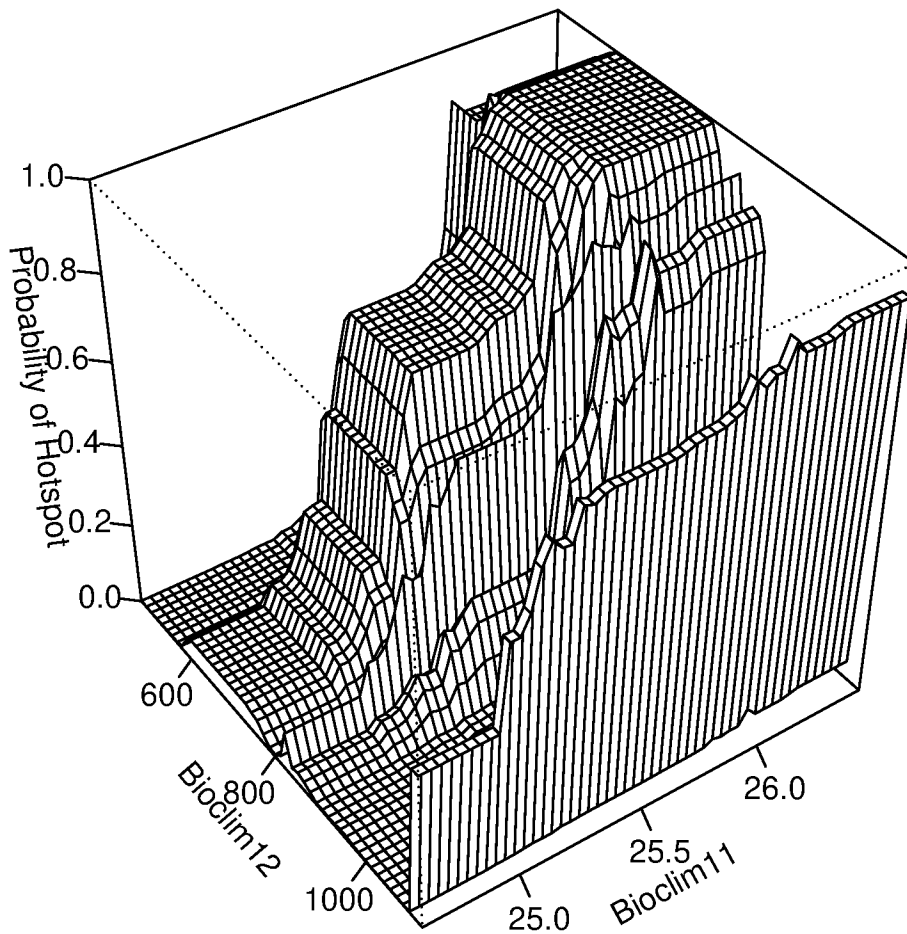

Supplement: S5 Fig — This figure depicts the strongest pair-wise interaction in the boosted regression tree model for village hotspot identity, between cluster 1, represented by annual precipitation (Bioclim 12), and mean temperature of the coldest quarter (Bioclim 11). In this three-dimensional plot the vertical z-axis represents probability of hotspot identity. (PDF) [file pntd.0008620.s010.pdf]

A

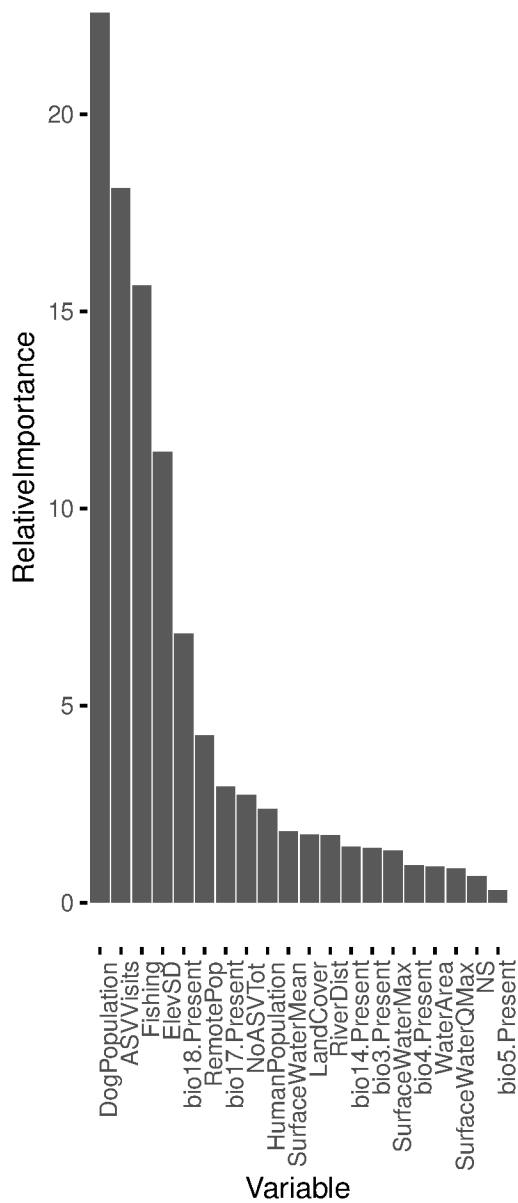

B

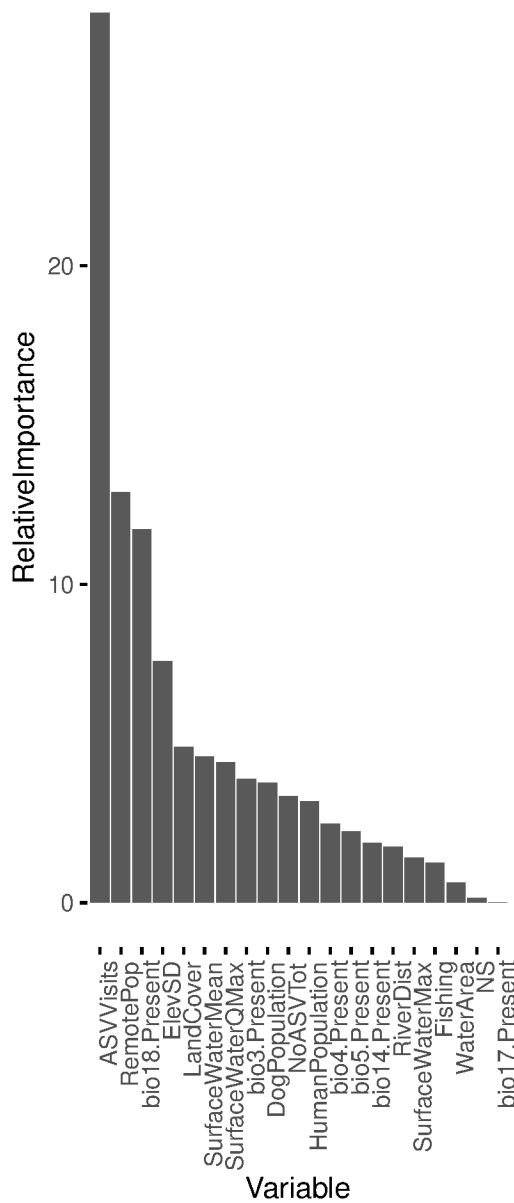

Supplement: S6 Fig — This figure depicts relative importance of covariates in predicting (a) D. medinensis presence and (b) hotspot identity. Unlike the main analysis, here models are trained on present climate data with a coarser spatial grain than WorldClim data. (PDF) [file pntd.0008620.s011.pdf]

A

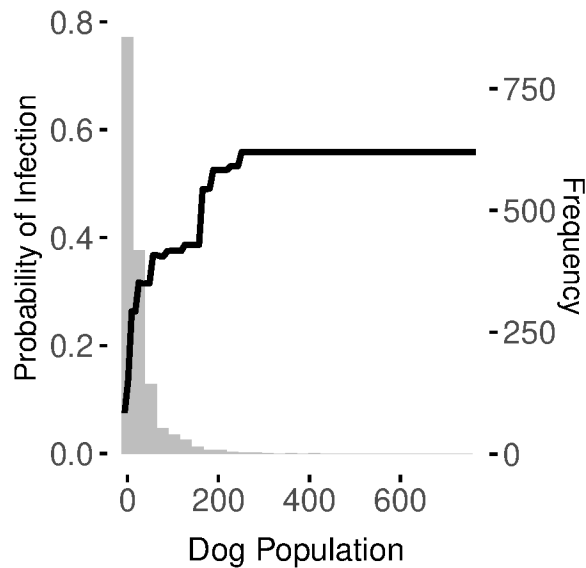

B

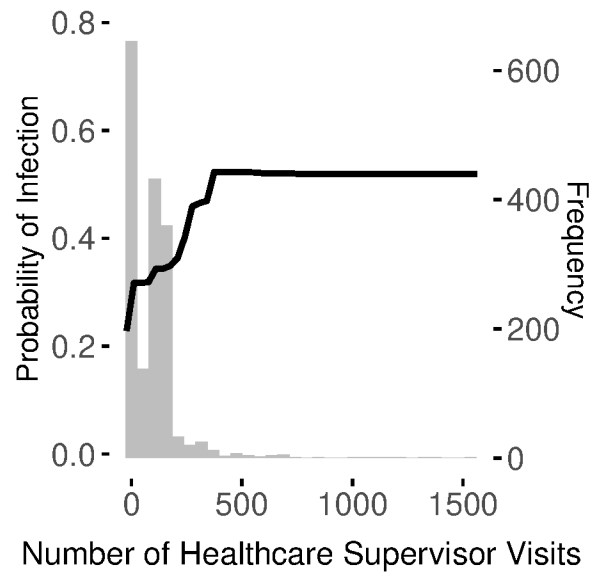

C

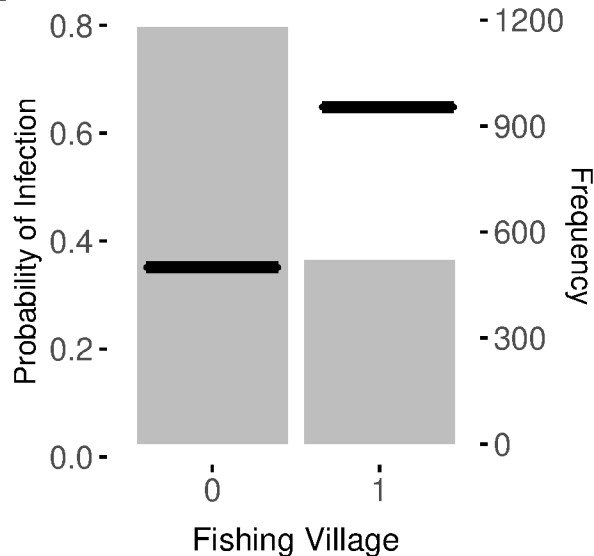

D

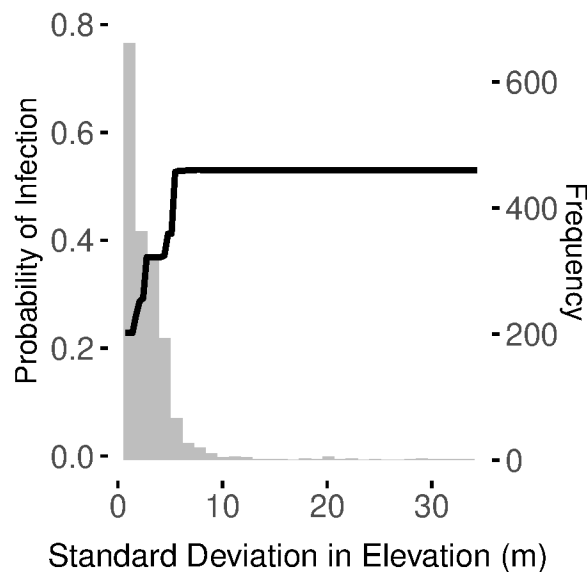

Supplement: S7 Fig — This figure depicts partial dependence plots showing the effect of (a) dog population, (b) number of healthcare supervisor visits, (c) fishing village identity, and (d) standard deviation in elevation on probability of parasite presence in all villages when present climate estimates are used rather than WorldClim variables. Histograms represent the distribution of values for these covariates amongst all training villages. (PDF) [file pntd.0008620.s012.pdf]

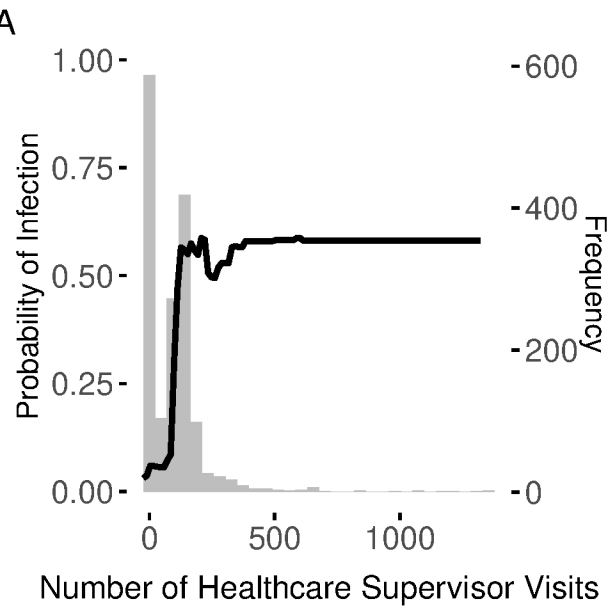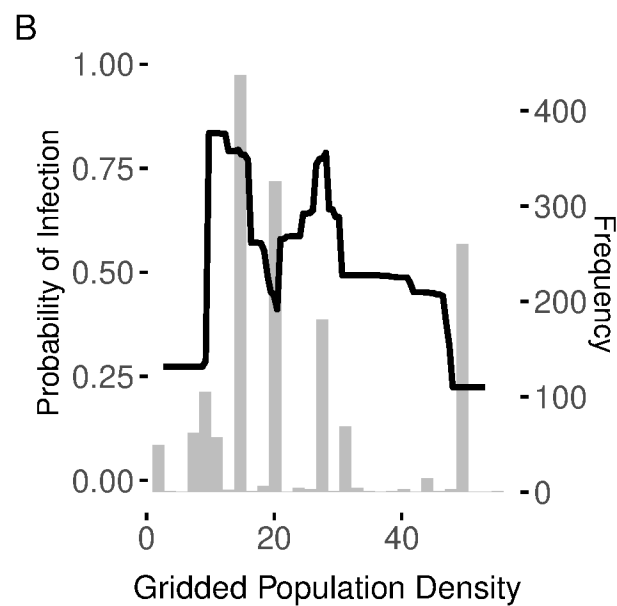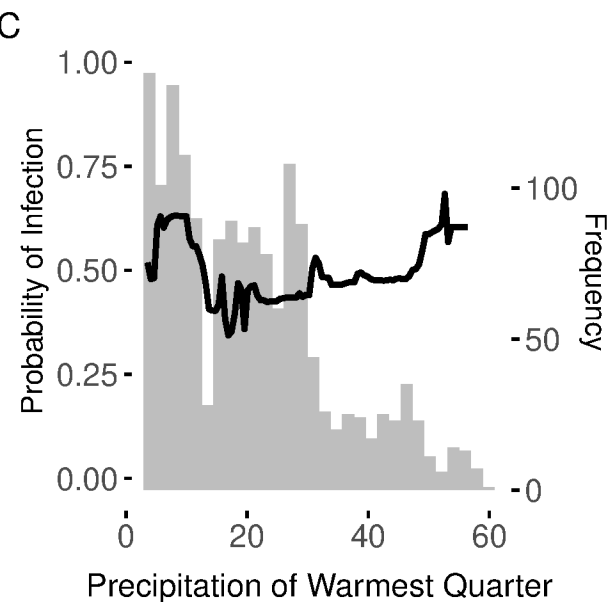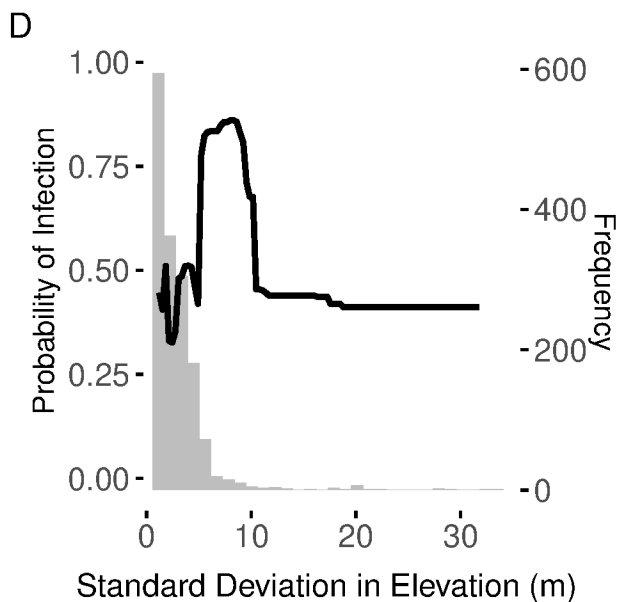

Supplement: S8 Fig — Partial dependence plots showing the effect of (a) number of healthcare supervisor visits, (b) gridded population density, (c) precipitation of the warmest quarter, and (d) standard deviation in elevation on probability of a village being part of a spatial infection hotspot. Histograms represent the distribution of values for these covariates amongst all training villages. (PDF) [file pntd.0008620.s013.pdf]
