# Supplementary material for: Identifying correlates of Guinea worm (Dracunculus medinensis) infection in domestic dog populations
Source: PLoS Negl Trop Dis. 2020 Sep 14;14(9):e0008620. doi: 10.1371/journal.pntd.0008620 (PMC7515199; doi:10.1371/journal.pntd.0008620)
Supplement: Attachment — Submitted filename: Response to reviewers_V3.docx [file pntd.0008620.s019.docx]

Re: Identifying drivers of Guinea worm (Dracunculus medinensis) infection in domestic dogs

Dear Editor,

We appreciate the opportunity to revise and resubmit this manuscript. We have altered the analyses and text according to reviewer comments and believe this has substantially improved the manuscript. Please find our response to individual reviewer comments below.

***Methods***

Reviewer #1: L132 ‘We obtained data on D. medinensis incidence in domestic dogs…’

Should the term ‘incidence’ be replaced with ‘cases’?

If incidence is the correct term, it would be helpful to know more information on how it was calculated given incidence requires knowledge on the population at risk e.g. was the population defined as dogs from the villages studied in this investigation? Given dogs less than 10-14 months old can’t be GW positive, does the population include puppies? Also, is a dog that has multiple worms counted twice?

***Changed incidence to “infections” to match the rest of the text and the methods. We count individual worm infections rather than infected dogs but the distinction is ultimately irrelevant to our analysis given that we summarize to presence-absence at the village scale.***

L141-142 ‘…but the number of infections per village varied widely (e.g. 2016 range: 0-71), and generally increased over time (Supplemental Fig 1).’

Given dog infections were only realised in ~2012, is this increase due to the increased surveillance effort for dog cases over time?

***The increase is likely due to both processes and we clarify this in the text (ll. 150-151). The addition of surveillance variables to our models also helps to control for this spatio-temporal change in surveillance effort. (ll. 172-175)***

What supplementary figure is this referring to? S1 is a table and I can’t work out which figure it would be, perhaps it wasn’t uploaded?

***This reference remained from a prior version of the text and has been removed.***

L147-150 ‘Finally, given recent work on the genetic structure of Guinea worm in Chad identifying Northern and Southern parasite sub populations…’

This is a neat idea. However, I can’t find any mention in the methods as to how this division was made? Were clear criteria used to determine this variable for villages? Some clarity on how this was done would be useful.

***We agree that this has not been sufficiently explained and attempt to clarify in the text that the prior genetic analysis identified a clear spatial boundary between two genetically related subpopulations (ll 156-159). This justified our explicit inclusion of spatial structure in our analysis and is further explained in Thiele et al. 2018 (cited in text).***

L151-152 ‘…we collated data on 32 environmental and demographic variables…’

Has there been any assessment for the models being overfitted or for collinearity?

I would want to see a correlation matrix for the predictor variables, or at least some report of the absence/presence of collinearity. I suspect that there is strong multicollinearity. If so, caution should be taken as even BRT models are not safe from the effects of strong collinearity (see Dormann et al. "Collinearity: a review of methods to deal with it and a simulation study evaluating their performance." Ecography 36.1 (2013): 27-46).

***We now present our analysis following variable reduction to control collinearity. We use Ward clustering based on the spearman correlation matrix as recommended by Dormann et al. and identify two co-linear clusters (see edits in text, ll 210-213; 265-269). The results do not change substantively (motivating our prior choice to present the simpler analysis) but we agree that this is the more appropriate analysis to present. We’ve also added language to clarify that we tune model parameters through 10-fold cross validation to test for and limit overfitting (ll. 233-235). The good congruence between CV and evaluation AUCs also implies lack of significant overfitting (ll 270-271; 276-277; 290-291)***

L 156-157 ‘…a single summary value, for the 6-year study period, was included for each village.’

What is the summary value (max, mean, median)?

***Edited this sentence to clarify that we use mean value. (l. 165)***

L165-166 ‘…lower spatial resolution data collected via remote sensing over the same time period as the parasite observations.’

What is the source of this remote sensing data?

***Reference to the source of the remote sensed data has been added to the main text (ll 181-184) and the supplementary data table.***

L174 ‘Spatial hotspot analysis…’

How was the location of the villages collected, via the GWEP staff?

More importantly, did the spatial analysis require a projection, if so what projection was used? In the Rcode in the supplementary material EPSG:26978 is used. However, this projection is for Kansas south and not Chad. This could lead to distortions that could impact any spatial analysis.

***Thank you for catching this coding error. Data were collected by Guinea Worm Eradication Program staff using a handheld gps in the center of each village. In order to extract linear distances to permanent water sources using the Hydrosheds database the village coordinates and Hydrosheds shape files had to be reprojected to the same system. This has been corrected to a more appropriate one (epsg:32633) but does not substantively affect our results.***

L187-191 ‘…(i) predicting whether D. medinensis was detected in a village over the 6-year study period and (ii) predicting whether…’

This sentence is very helpful as it clearly states the purpose of the BRTs and mentions an important consideration of villages in hotspots. In fact, this whole paragraph for BRT methods is very well written.

My only criticism is that AUC is not mentioned here and yet it is a key part of the reporting in the results (L224).

***We add the use of AUC as a method of measuring model fit to this paragraph (ll 230-233).***

L191-194 ‘…we also (i) allowed interactions between longitude/latitude and other covariates…’

(1)	Were interactions allowed between collinear predictors?

(2)	If surveillance effort changed over the course of the observation period, could this be accounted for in the BRT models?

(3)	There is also the effect of control measures that could influence the status of a village. Again, can the BRT models account for a measure of variation in control strategies used in villages e.g. abate might be more aggressively/frequently applied in some areas than others.

I realise that 2 & 3 might be hard to include in models, but they could be very important. It would be good to at least acknowledge them as caveats in the discussion.

***1)  See above for our change in the analysis to control for collinearity of predictors. We believe that this solves the first concern***

***2/3) Surveillance effort has certainly changed over time and space. As mentioned, this is somewhat difficult to control for with the data collected. We simplified our analysis to simple presence/absence over the period of the study (rather than including a temporal aspect or attempting to predict number of infections) to limit the influence of this temporal bias (ll 151-155). In order to better control for the spatial bias in surveillance we include two newly sourced covariates for the mean number of healthcare workers/year in a village and number of visits from healthcare supervisors over the period of study. The second variable encompasses some degree of spatio-temporal variation in surveillance as villages have no visits for years that they went unsurveilled. We understand this to be a reasonable, though far from perfect, measure of intensity of both surveillance and intervention. Number of healthcare worker visits accounts for a substantial amount of variation across most models but does not substantively alter the identity of other important variables (ll 172-175; 272-274; 291-294). We should note that these new healthcare worker data only extend back to 2013 so we have removed records from 2012 from our analysis. This results in only 6 villages being reclassified as guinea worm free which were previously guinea worm positive due to infections in 2012.***

Reviewer #2: Yes the study desing is appropriate to unearth factors predicting endemicity of Gunea worm infection. The population  of villages and corresponding number of dogs was also appropriate. Appropriate discussion is presented as to the limitations that may arise due to sample size. The statistical tests performed on the data was also satisfactory as was the conclusions drawn from them. No ethical concenrs are required for these studies.

Reviewer #3: See below. There are issues with the confounding effects of changes in disease surveillance that are likely happening at the same time as the animal epidemic is increasing. It will be hard, if not impossible, to disentangle these. There are other smaller issues, e.g. how fishing villages are classified.

***See response to comments below. We attempt to clarify in the text the way our analysis minimizes the effect of increasing surveillance through time and add to our  analysis a control for spatial variation in surveillance.***

--------------------

***Results***

Reviewer #1: L209-217 ‘3.1 Identifying spatial hotspots of infection…’

I find this entire section very confusing. Some of the numbers do not add up (see my comments below) or, if they do, require some explaining.

In addition, I think some small adjustments could help to better guide the reader through the results. For example, rather than reporting the number of ‘GW positive villages / total villages’ in the methods (L140), it would flow better if put in the results. I would then want to know how many villages were identified in hotspots and, within this, how many were GW positive and GW negative villages. This would help to identify how many GW positive villages have not been identified in hotspots and how many GW negative villages are included in hotspots. It would then be intuitive to provide the same break down for the north and south area.

***This section has been substantially rewritten to take these comments into account. We clarify in the methods that the number of villages included in the hotspot analysis is slightly smaller than the total number of villages due to missing data (ll 197-199). We move the summary statistics about infections in villages to the beginning of the results section(ll 245-247). We also reframe the descriptive statistics about hotspots to ignore the train-test split as this causes confusion in the numbers and is irrelevant to this section. We’ve also added the proportion of hotspot villages containing an infection (ll 249-257).***

If possible I would also like to see the sample size/means ± SE for the data on villages that is used in the BRT models. My suggestion would be to either provide a new table or to add this data to table S1 (with a column for all villages, a second for northern villages and a third for southern villages). Maybe just for the WorldClim data?

***Means and SEs have been added to table S1 for all appropriate variables along with sample sizes for overall, north, and south. Note that these do not perfectly line up with the sample sizes in the paper because these summary statistics are of all data prior to the train/test split and analyses were conducted after the train/test split.***

L211-212 ‘Overall, 44.2% (703 out of 1592) of villages were identified as belonging to a spatial hotspot (Fig 1)…’

Wasn’t there 2125 villages (reported in the methods L135), where has 1592 come from?

***As more generally described above, the number 1592 comes from the loss of villages in the hotspot analysis to missing data in the dog population of 135 villages (to which the BRT is robust) and the division of our data into a 80-20 training testing split. This has been substantially simplified. More explanatory text has been added to the methods (ll. 245-247) and results, and the train-test split has been removed from this portion of the results as it is irrelevant. (ll 249-257)***

L212-214 ‘… 53.9% (637 out of 1182) of villages belonged to a hotspot, with fewer clusters in the southern/eastern reaches where only 16.1% (66 out of 410) of villages belonged to a hotspot.’

The totals in the brackets sum to the total mentioned in L211 (1592), but again I’m not sure where this total has come from. Am I right in thinking the 1182 and 410 is the total number of villages studied in the north and south respectively?

***We agree that this was a confusing way of formatting these summary statistics. Your thinking is correct but we have chosen to reframe this summary as the proportion of all hotspot villages in each location, as compared to the proportion of total villages in each location. (ll 249-257)***

L224-225 ‘The BRT model fit data on D. medinensis presence across all villages with a mean AUC of 0.895 in cross-validation and an evaluation AUC of 0.897.’

As mentioned in a previous comment, it would be helpful to introduce the use of AUC in the methods.

***The description of AUC has been added to the methods above (ll. 230-233)***

L232-235 ‘Fishing village identity was only important in northern villages (as suggested in Fig 3c)…’

Mentioning Fig 3 here is misleading as it could be interpreted as this figure showing data for the analysis on just the northern villages (when it is for the analysis on all villages).

***The reference was intended to draw a connection between the interaction in figure 3c and the north/south analysis. Following variable reduction the reference is no longer relevant and has been removed rather than clarified.***

L246-249 ‘…although there were many important interactions, the largest of which was between latitude and mean diurnal temperature range…’

It isn’t clear to me what the interaction is between latitude and mean diurnal temperature? I don’t think this is mentioned in the discussion and so it isn’t clear why this is important? Also, why is no comment made on these other important interactions? There are some further interacting variables with longitude, do they not hint as to what is going on in the southern villages?

***This section has been amended to match the results of the collinearity controlled analysis and the reference to extraneous interactions removed.***

L254-257 ‘Precipitation of the coldest quarter emerged as important in predicting dog infection at the village scale (10.2% Importance). This change…’

This point would be clearer if the actual change was mentioned.

***We agree with this note but the distinction is no longer relevant with the variable reduced analysis.***

Figure 1

This might produce an ugly figure, but if possible it would be great if some/all of the following could be identified in the map:

(1)	The 14 spatial hotspots. It would help the reader gauge the appropriateness of the spatial scan and provide visual information on the spatial clusters (i.e. their distribution and variation in size).

(2)	The north/south divide

(3)	GW positive & negative villages

***We have redesigned the figure in line with these suggestions. Points represent positive and negative villages, hotspots are denoted by circles, and the N/S divide line is also inserted.***

Figure 2

Nice plots, just need to add the panel labels (A, B, C, D).

***Thank you! Letter labels now added to all plots.***

Figure 3

Need to add the panel labels. I would also suggest changing the y axis for the ’probability of infection’ so that they are the same in each plot.

Are the histograms for the frequency of GW positive villages or for all villages? This should be clearer in the figure description. I assume (given the numbers presented) that this is for all villages. If true, would it not be more informative in the context of these plots to show the frequency of GW positive and negative villages? Currently it is hard to see if the model is a good fit to the data.

***Letter labels have been added to all panels of all plots. Y axes of all partial dependence figures have been standardized to be consistent across panels in a single plot. Your interpretation of the histograms is correct, the histograms represent all villages, and the figure captions have been amended to make this more clear. While including bars for both presence and absence villages may be informative, we have experimented with this and feel it complicates the figure to the point of being difficult to interpret.***

S1 Table

Make sure that the names of variables are consistent with the plots for relative importance e.g. in the table there is 'Distance to nearest permanent water', but this does not appear in the plots for relative importance. I assume that 'RiverDist' is the equivalent variable?

***The table has been updated to more clearly align with variable names in figures.***

Reviewer #2: Yes the analysis matches the pan and results are clearly presented. I found the figures to be reasonably presented. Figures 3 and 4 could use a bit clearer labels on y axes.

***The axes of figures 3 and 4 have been rescaled so that all panels match across a figure. We would be happy to label the axis differently but are unsure what would make the labels more clear.***

Reviewer #3: See below. Some of the results are interesting and informative, e.g. the interaction between fishing status and longitude and the variation in elevation. I have reservations about the other results.

***See below for more extensive response. We have altered the text to better explain the role of dog population in our models and emphasize the results most potentially relevant to control. We have also included controls for surveillance bias in our analysis.***

--------------------

***Conclusions***

Reviewer #1: (No Response)

Reviewer #2: Yes to all these points.

Reviewer #3: See below. The conclusions in relation to targeting and control and poorly supported by the analyses.

***See below for our discussion of how we have addressed these concerns in the analyses and text.***

--------------------

***Editorial and Data Presentation Modifications?***

Reviewer #1: (No Response)

Reviewer #2: (No Response)

Reviewer #3: L94 – in relation to the classical (typical) life cycle, presumably, the ingestion of copepods in water while drinking applies to humans and to other mammalian hosts, as much as the non-classical cycle mentioned in the next line.

***Edited this sentence to clarify that drinking of unfiltered water is a potential mechanism of transmission for all hosts. (ll 99-102)***

L97 – there is a “potentially” missing here, to read “potentially allowing them” since it is not clear that they do act in this way to humans or other hosts.

***This is a good point. The sentence is revised to clarify the uncertainty in this mode of transmission. (ll 99-104)***

L106 – yes the number of dog infections recorded has increased, though it is not clear that this is due to a genuine increase in incidence or to an improvement in recording.

***The particulars of the interaction between increased surveillance and possible increase in parasitism are now more fully addressed in the methods (ll 150-151). This sentence has been revised to clarify the point that control mechanisms do not seem to be decreasing infections in dogs in the same way they have in humans (ll 111-113).***

L135-136. Is surveillance really a ‘daily search’ across >2000 villages? Or rather an elicitation of case reporting, via rewards schemes and a reporting network? This description suggests something much more labour intensive, comprehensive and systematic than the latter.

***Your presumption is correct. We’ve corrected the description to more accurately represent the reporting scheme (ll 141-146).***

L142 – Figure S1 and Figure S2 were not included in the review manuscript and were not available for download.

***These references are a remnant of a prior draft and have been removed from the text.***

--------------------

***Summary and General Comments***

Reviewer #1: The authors present a village/regional level analysis of the environmental and anthropogenic predictors of Guinea worm infection in dogs in Chad. The most striking result suggests there are different drivers of infection in northern and southern villages. In the north, ‘fishing’ villages have a higher probability of infection, while environmental variables seem more predictive of cases in the south. These results are important for informing control efforts of an eradication program that has entered its final stages.

Overall, this is a well written manuscript on a very topical and interesting subject. However, I think there are several areas for improvement/clarity that need to be made before the manuscript can be accepted for publication:

(1)	The analytical methods are appropriate and articulated clearly, but there is no mention of any checks for model overfitting or collinearity of the predictors. Collinearity could cause problems in the interpretation of the BRT model output. If strong collinearity is discovered and the models need to be altered, the results could be substantially changed given that BRT models are sensitive to even small perturbations of the data input.

***As discussed above in response to specific comments. We now present an analysis that directly addresses collinearity (ll 210-213; 265-). We also clarify in the methods how our parameter tuning serves to limit overfitting (ll 265-269).***

(2)	There needs to be some presentation of the sample sizes for the variables being used in the BRT models. This would help the reader to get a better understanding of the data being analysed.

***As discussed above, the text describing the numbers of villages identified in the hotspot analyses has been clarified (ll. 245-247). And the total sample sizes for boosted regression tree analysis are added to the methods (ll 213-218).***

(3)	Some of the results do not align with that mentioned in the methods and need to either be corrected or an explanation provided. There is also the scope to provide more summary information that would be useful to the reader.

***While we are not entirely clear on what is referenced here we have attempted to provide more summary information, where appropriate, and to clarify the connection between methods and results, particularly with respect to the hotspot analysis. These changes are detailed above in response to more specific comments.***

Reviewer #2: (No Response)

Reviewer #3: The eradication of Guinea worm has progressed well and animals are now a barrier to further progress. The topic of this paper is therefore of considerable interest and is ideally suited to this journal. Understanding the biological drivers of Guinea worm infection/incidence in animals and dogs in particular would be valuable to future control efforts. However, I have several reservations about the specifics of this analysis, which, in my view, limit the utility and relevance of this contribution.

In their summary, the authors offer the results as guidance for targeting control and surveillance, and point towards elevated risks associated with whether villages are engaged in fishing, larger dog populations, longitude, variation in elevation. They also analyse village location in hotspots. Of the effects reported, variation in elevation and, possibly fishing activity (if this is independent of survey methods and variation in surveillance intensity - see below), are potentially the most biologically informative. However, the other effects might alternatively be framed as recommendations to look for dog infections where there are lots of dogs and, if surveillance effort follows infections, to look for more infections where surveillance has been focused previously. Similarly, the effects of longitude are hard to translate into guidance, other than in relation to fishing, and presence in a hotspot appears compromised by the overwhelming effect of apparently categorising

the NW Chari into a single hotspot. I detail concerns about each of these areas in the following points.

***We have attempted to address each of these concerns either through changes to our analyses or the text. See below for specific discussion of each case.***

There is no mention of variation in surveillance, which presumably has varied spatially and temporally over the duration of the study and since the re-emergence of Guinea worm in Chad. It is important to quantify how surveillance might have changed and to take this into account. A glance at the detail of Guinea worm surveillance activities in the “Guinea worm wrap-up” suggests that surveillance has been stepped up across the board in Chad: “Some of the increase in infected dogs reported probably resulted from expansion of the number of villages under active surveillance (VAS) from 1,895 at the end of 2018 to 2,138 as of May 2019.” Villages have also apparently been classified as Level 1 to Level 3 villages, presumably reflecting varying surveillance effort. Which villages were subject to the greatest surveillance and how did this vary over time as both the apparent epidemic and surveillance were growing? Are the authors actually demonstrating the effects of an increase, or

spatial intensification, in surveillance effort. For control purposes, this variation in effort might very sensibly be focused on big villages, fishing villages, those in particular regions, or in infection hotspots (as in Levels 1-3). It is important to isolate and account for these obvious and influential logistical, practical changes in surveillance as a driver of variation in the detection of infections, as much as genuine biological risk factors. Either way, these potential effects cannot reliably be described as drivers of infection in an epidemiological sense.

***Surveillance effort has certainly changed over time and space. As mentioned this is somewhat difficult to control for with the data collected. Surveillance “Level” is a classification system based entirely on the prior presence of guinea worm in a village or neighboring villages. As such all GW positive villages in our analysis are classified as the highest surveillance level, making it an unhelpful and circular classification to control for surveillance effort. We simplified our analysis to simple presence/absence over the period of the study (rather than including a temporal aspect or attempting to predict number of infections) to limit the influence of this temporal bias (ll 151-155). In order to address these comments and better control for the spatial bias in surveillance we include two newly sourced covariates for the mean number of healthcare workers/year in a village and number of visits from healthcare supervisors over the period of study (ll*** ***172-175; 272-274; 291-294). The second variable encompasses some degree of spatio-temporal variation in surveillance as villages have no visits for years that they went unsurveilled. We understand this to be a reasonable, though far from perfect, measure of intensity of both surveillance and intervention. Number of healthcare worker visits accounts for a substantial amount of variation across most models but does not substantively alter the identity of other important variables (ll 272-274; 291-294). These variables are not co-linear with other covariates in the models, but see below for a specific discussion of fishing village identity and surveillance intensity, but their inclusion should account for any variation currently ascribed to other variables that is actually a result of variability in surveillance. We should note that these data only extend back to 2013 so we have removed records from 2012 from our analysis. This results in only 6 villages being reclassified as guinea worm free which were previously guinea worm positive due to infections in 2012.***

The effect of dog population size on the classification of a village as an “infected village” is not at all surprising, and would be apparent if the incidence of infection was genuinely uniform across all areas. Indeed, it would be surprising if this were not an effect. This “per village” rate of infection, is not to be confused with an effect of dog population size on the “per dog” rate of infection, though the latter has not been quantified or tested. This is likely to be the unavoidable consequence of being more likely to find a dog infection when there are more dogs to look at. The authors acknowledge this at L276. However, it would clearly be a mistake to interpret this as a driver of infection and/or to target surveillance or control on this basis. The distribution of dog population sizes in Figure 3 suggests that almost all villages have small dog populations, perhaps <~100 dogs and so many small villages would still harbor dog infections if surveillance was focused

upon the few, larger villages.

***We agree with this analysis and had no intention of suggesting otherwise. As such we really appreciate the comment that we have been unclear on this point. We have attempted to amend the methods and discussion sections to clarify this point (ll 165-169; 325-331). We have in particular removed pointed suggestions of targeted surveillance on villages with high dog populations as we acknowledge this logic to be inherently circular.***

Similarly, the longitude effect, which is the largest single effect across the paper, is not clearly spelled out and so the biology and targeting consequences remain unclear. According to Figure 1, the Chari River runs N-S, but the southern affected area lies to the E and the Northern Area to the W. If we look at Figure 1, this maps out villages in hotspots and those that are not, but not the hotspots themselves. There are no green dots north of about half-way up the country. So are all the orange dots in the North, i.e. all the infected villages in the north, all in one big hotspot, and the others all in very much smaller separate hotspots? The text suggests there are 14, but 14 are not apparent from the figure. The Figure is also cut off at the bottom and would benefit from lines of longitude. Presentational issues aside, the differing magnitude of these hotspots seems likely to introduce a heavy influence of this single large NW hotspot in later analyses. Thus, the

analysis reported at L250-257 and in Figure 4 appears largely to distinguish the location of the “NW big hotspot”, i.e. all the villages in the NW, to be in the NW hotspot. Again, this does not appear helpful and would be misleading in targeting.

***While we agree that this comment identifies a weakness in the discussion section this section has been substantially rewritten as a result of the co-linearity controlled analyses. While the cluster containing longitude and latitude remains important in predicting hotspot identity, healthcare worker visits now explain a lot of this variation as well. Due to the co-linearity between location and many other variables we are forced to discuss the importance of geography and climate in a more general way (ll 385-397). We agree with the likely dominance of the large North-West Hotspot and now acknowledge such in the text (ll 395-397).***

L159-162 It is not made clear how villages were classified as a “fishing village” or otherwise, or how the “majority of the population” was determined? According to the supplementary information, this was done by the Guinea worm survey field teams, it is therefore possible that this introduced a systematic bias with respect to Guinea worm incidence. This needs to be shown to be otherwise. That said, the interaction between fishing village classification and location in the NW or SE is potentially of interest.

***This is a good point as to the robustness of our methods to surveillance bias. While the core issue of fishing village status being identified by GWEP workers on the ground on a case by case basis cannot be remedied, we provide in the text the clearest description of the quantitative threshold of majority of families fishing used by the surveillance officers. We have also attempted to address the spatial bias in surveillance intensity by including two metrics of healthcare worker surveillance (mean number of healthcare workers resident in a village and number of healthcare supervisor visits over the 5 year period) (ll 172-175). We also assessed whether fishing villages on average receive more surveillance than non-fishing villages. While fishing villages (mean = 107.9) do on average have more healthcare supervisor visits than non-fishing vilages (mean = 88.9) the difference was not significant according to a Wilcoxon rank-sum test (p = 0.47). Fishing villages (mean = 10.9) and non-fishing villages (mean = 8.3) differ significantly in the number of resident healthcare volunteers (p = 0.01) but our inclusion of these variables in our models is designed to control for this difference. Resident healthcare workers was never a highly important variable in analyses as we would expect it to be if it better accounts for the variation in disease presence accounted for by fishing village status.***

L262 it is not elevation, but standard deviation in elevation that was influential. This is potentially an important distinction, given the variation in relief associated with surface water availability.

***We have gone through the text to make sure all references to standard deviation in elevation are clear.***

Incidentally, the analyses appear not to distinguish between villages in which one infection has been recorded and those in which infection is a more frequent occurrence? It appears as though the analysis distinguishes between a binary classification. This seems like a missed opportunity, given the availability of data and the very wide variation in reported cases over the study.

***This interpretation of our analysis is correct. We chose to pose the question this way (i) as a way of controlling for variation in surveillance through time for which we are unable to directly control and (ii) because this more closely addresses the major public health issue addressed by our colleagues working in the eradication program: identifying entire villages not currently infected which are likely to become infected. We’ve attempted to clarify this in the text (ll 154-160).***
