# Supplementary material for: Identifying correlates of Guinea worm (Dracunculus medinensis) infection in domestic dog populations
Source: PLoS Negl Trop Dis. 2020 Sep 14;14(9):e0008620. doi: 10.1371/journal.pntd.0008620 (PMC7515199; doi:10.1371/journal.pntd.0008620)
Supplement: Attachment — Submitted filename: GW BRT Second Response To Reviewers.docx [file pntd.0008620.s020.docx]

**Response to Reviewers**

**Methods**

Reviewer #1:

*Reviewer:*

*L161 – 164 ‘…we collated data on 35 variables that might contribute to transmission….’*

*There are still some inconsistencies and areas for clarification in the use of language in the methods, Table S1 and Figure 2. See my specific comments for Table S1 and Figure 2 below.*

*An example is ‘RemotePop’ which appears in Figure 2. From the results it looks like this is a measure of the population from remote sensing data? However, there is no mention of this variable (or how it is calculated) in the methods, and it is not in Table S1.*

*Aren’t there more than 35 variables considered when you include the controls for surveillance effort?*

**Response:**

**RemotePop is in face remotely sensed population and has been added to Table S1. Abbreviations have been added to this table where absent. There are 36 variables and this has been corrected. We have fixed these inconsistencies among the text, Table S1 and Figure 2. Apologies for the confusion.**

*Reviewer:*

*L172 ‘Villages were identified by GWEP staff as “fishing” if the majority of families...’*

*Be more explicit, what is meant by majority, >50%?*

**Response:**

**Yes. The text has been corrected to read *“if greater than 50% of families in that village fish”* (ll.166-167 in tracked changes document)**

*Reviewer:*

*L173 - 176 ‘To control for spatial variation in surveillance, we also included two measures of surveillance effort…’*

*Add a few words to explain the differences in what these control for, it is not immediately clear. Perhaps just add a line to the same effect of that in your reply to reviewer comments ‘The second variable encompasses some degree of spatio-temporal variation in surveillance as villages have no visits for years that they went unsurveilled.’*

**Response:**

**Great point. The following sentence has been added :**

***“These two metrics capture different components of surveillance intensity: the first variable summarizes spatial variation in average surveillance intensity over the study period, while the second variable encompasses some degree of the spatiotemporal variation in surveillance since villages have no visits for years during which they were unsurveilled.****”* **(ll.188-194 in tracked changes document)**

*Reviewer:*

*L211 – 213 ‘…we diagnosed significant co-linearity in predictors…’*

*Did you include the variables for sampling effort in the co-linearity analysis? It would be interesting to know whether or not measures of sampling effort are correlated with demographic parameters.*

**Response:**

**We did include these surveillance variables in the co-linearity analysis. They were not significantly co-linear with any other covariates (including each other), which is interesting but outside the scope of the paper. We’ve added *“all types of predictors”* to the above sentence to clarify this. (l. 215 in track changes document)**

*Reviewer:*

*L213 – 214 ‘Co-linear clusters were reduced to the single most central variable…’*

*My understanding is that this method involves omitting variables identified in a cluster (except the central variable) from the model. With this in mind, why do variables in the clusters (that are not the central variable) appear in the relative importance plots and interaction tables? For example BIO9, BIO11 and remotely sensed population. Is this because you only omitted variables in a cluster if they went above a threshold e.g. r > 0.7?*

**Response:**

**Thank you for pointing this out. This, like the issues with Table S1, is a result of mis-entered data in the table rather than an issue with analyses. The clusters listed in Table S3 were incorrect and have been corrected. You will now notice that the only variables in the analysis are those that are not in a cluster or a central variable of a cluster. Also note that clusters of presence climate variables differ from the main analysis. We have added table S5 to display the clusters for the supplementary present climate analysis.**

*Reviewer:*

*L224 – 227 ‘…we also (i) allowed interactions between longitude/latitude and other covariates…’*

*Why are interactions added into the models when they are ignored in the results and discussion? Furthermore, it isn’t entirely obvious to me how to interpret the interaction results reported in the supplementary tables and figures. I would expect that these interactions are quite important to our understanding and interpretation of the results and therefore some elaboration is required.*

**Response:**

**Interaction depths of our models are tuned to improve fit of the model. While these interactions can be inspected/interpreted we do not find that they change the qualitative understanding of the role of these variables in contributing to our response variables. We include a visualization of the strongest interaction for the presence-absence model and the hotspots model as Figures S13 and S14. The interaction between Bioclim 12 (cluster 1) and Bioclim 11 in the hotspot model (Figure S14) was overwhelmingly the strongest interaction in any of our analyses. The visualization shows the lack of additional qualitative information provided by this interaction. The surface produced looks largely like the shape of the partial dependence curve of Bioclim 12 rescaled according to the shape of the partial dependence curve for Bioclim 11. The main departure occurs at very high values of Bioclim 12 where the shape of the effect of Bioclim 11 differs observably from the shape at intermediate levels of Bioclim 12. This difference could be characterized as along a continuum from step-like increase to smooth increasing curve. While perhaps interesting, this result does not change our interpretation or discussion of the model output and we relegate it to the supplement to avoid the (already somewhat complex) manuscript becoming too complicated. Find the rough partial dependence plot for Bioclim 12 and 11 below.**

**We add the following text to clarify:**

***“Bag fraction denotes the size of the random sample on which each tree is trained, learning rate sets the shrinkage of the contribution of a given tree to the overall model, and tree-complexity defines the number of branch splits allowed in the tree. Tuning for tree complexity requires fitting interactions between covariates. The strength of the interactions and a partial dependence plot of the strongest interaction for the presence-absence (Table S2, Figure S13) and hotspot (Table S6, Figure S14) models are reported in the supplementary material since they do not provide information that qualitatively changes the interpretation of the non-interaction based results”* (ll. 239-246 in tracked changes document)**

*Reviewer:*

*Also, I interpreted this to mean that separate N/S analyses were done for both the village and hotspot scales. However, this does not seem to be the case. I would suggest either adding the N/S analyses for the hotspot scale or to be more explicit and to say that this analysis was only conducted for the village scale.*

**Response:**

**We have added supplementary Figure S12 showing the results for the N/S split analysis at the hotspot scale. The results are not appreciably different between the north and south or between either and the overall analysis. Also added the following text to the results:**

***“These findings were not qualitatively different when analyses were performed on northern and southern villages separately (Figure S12).”* (ll. 308-309 in tracked changes document)**

Reviewer #3:

*Reviewer:*

*In relation to objectives and design:*

*I think that in this revision there remains a mismatch between the prominently stated ambition to identify drivers of infection in dogs and the reality, which is identification of correlates of village-level detection of dog infections and a coarse analysis of locations of infection hotspots. I don't think this is being fussy. The authors just need to be a bit more honest and conservative in the headline elements of the paper when it comes to talking about what they are able to do with the data they have. Therefore, the Methods adopted are appropriate to a different aim of understanding correlative patterns in the long-term detection of dog infections in villages, but they are not suitable, and the data underpinning the analyses are not suitable, for the stated aim of an analysis of causative drivers of infection in dogs. I don't think therefore that it is possible to support the title or Abstract with the data used and Methods adopted with any amount of revision of the*

*approach. Rather the stated ambition, title and scope of the paper needs to shift to fit with the data, Methods and analyses.*

**Response:**

**We certainly take this point and have made edits to the title, abstract, and author summary (replacing “drivers” with “correlates”) in response to this comment and specific comments below to clarify that the analysis identifies highly statistically predictive correlates of the detection of dog infection in villages of Chad. We have also made additional edits to the discussion and introduction to match this shift in tone.**

*Reviewer:*

*I have no concerns about ethical or regulatory requirements.*

--------------------

**Results**

Reviewer #1:

*Reviewer:*

*L292 – 295 ‘The most important variables included cluster 1 (represented by Bioclim 12, 36.0% Importance)… mean temperature of the coldest quarter (10.2% Importance) and mean temperature of the driest quarter (Bioclim 9, 9.6% Importance).’*

*To help the reader there needs to be some consistency when referring to Bioclim variables. I would suggest using the format you use at the end of this sentence when referring to Bioclim9 i.e. say what it actually is and add the shorthand in brackets.*

**Response:**

**The references to bioclim variables have been cleaned up to consistently match this style of *“plain text description (Bioclim XX)”***

*Reviewer:*

*Figure 1*

*Can you provide a reason for why the spatial hotspots overlap? This doesn’t seem right to me and I would suggest that something has gone wrong, however, I do not know the intricacies of the spatial scan and whether or not this is normal behaviour. The obvious answer would be due to temporal overlap, but this cannot be the case in this analysis as GW positive villages are identified using the entire observation period. Is there something in the R package guidance or literature that could explain this, or is this an error? Perhaps the scan is using the temporal aspects of the data rather than ignoring it?*

**Response:**

**This is not an error. To quote from the satscan documentation “The standard purely spatial scan statistic imposes a circular window on the map. The window is in turn centered on each of several possible grid points positioned throughout the study region. For each grid point, the radius of the window varies continuously in size from zero to some upper limit specified by the user. In this way, the circular window is flexible both in location and size. In total, the method creates an infinite number of distinct geographical circles with different sets of neighboring data locations within them. Each circle is a possible candidate cluster.”** [**(Kulldorff 2017)**](https://www.zotero.org/google-docs/?smXXrB)

**This means that there are a number of possible radius clusters all centered on the same point (potentially resulting in concentric clusters). Clusters can also overlap when they have different centers because the algorithm selects clusters that comprise areas with significantly more outbreaks than expected. There is no inherent reason why these areas could not overlap. The following sentence has been added to the text to clarify:**

***“Because the spatial scan is performed at varying radiuses centered on all villages, overlapping or concentric clusters are possible”* (ll. 205-206 in tracked changes document)**

*Reviewer:*

*Table S1*

*A few suggestions to help guide the reader:*

*1)	Include the abbreviations used in Figure 2 in brackets or add them to the caption for Figure 2.*

*2)	Add RemotePop*

*3)	Add a section of rows for the variables controlling for sampling effort and report their mean & SE*

*4)	For the last three column headings keep the ‘N=’ but change the rest of the text to something like ‘All villages’, ‘Northern villages’ and ‘Southern villages’. Then, in the legend, put something to the effect of ‘Where appropriate the means (± SE) are reported’.*

*5)	Include the number and/or proportion of villages that were identified as fishing villages, rather than leaving it blank.*

*6)	My understanding of the choice of collinearity analysis is that many of these covariates were omitted from the model. If so, consider highlighting those that were used in the model by putting them in bold.*

**Response:**

**These are all excellent suggestions and the changes have been made.**

*Reviewer:*

*Table S2*

*This is not referenced in the main text. Furthermore the relevance of these interactions and how to interpret them should be discussed, currently it is not clear why they were considered in the first place or what they mean.*

**Response:**

**See discussion above about interactions (response lines 93-124). Interactions are included due to model tuning and these tables are now referenced in the methods. They do not provide qualitative information that would alter our results/discussion.**

*Reviewer:*

*Captions and legends*

*Check these carefully as some are not correct e.g. for Figure S7 it mentions panels a-d, but only two graphs are presented.*

**Response:**

**These have been double checked and the discrepancy in the caption for Figure S7 has been corrected.**

Reviewer #3: (No Response)

**Conclusions**

Reviewer #1:

*Reviewer:*

*L320 – 322 ‘Overall, our results suggest that…’*

*The final part of this sentence is a bit repetitive of the previous and doesn’t really add much. How can correlates with the broad hotspot scale help the GWEP?*

**Response:**

**We havedded the following text to the end of this sentence to clarify how correlates of hotspots may be informative for GWEP:**

***“suggesting that spatial epidemiology may be most appropriate for identifying infection risks at this broader scale”* (ll. 339-340 in tracked changes document)**

*Reviewer:*

*L383 – 402 ‘In addition to our village level analyses, the identification of spatial hotspots…’*

*I think this paragraph discussing the hotspot analysis could be improved. While the points raised are interesting, I think more needs to be said about the variables that have been identified to characterise a hotspot and how these can be used by the GWEP. The model suggests that hotspots might be predicted/characterised by the mean annual precipitation, and the correlation of this variable with a number of other variables is worth discussion. There were also interactions that were identified, however, as I mentioned earlier, many of these are with variables in the same collinear cluster and will not be present once removed from the model.*

**Response:**

**We have been asked by another reviewer to scale back the extent to which we claim these results are useful for GWEP as they are primarily correlative. We have rewritten parts of this paragraph with this comment in mind but know that we’re trying to balance these two very valid perspectives! Also see above (response lines 93-123) for further discussion of interactions.**

*Reviewer:*

*Why was the hotspot analysis not separated into N/S as with the village level analysis?*

**Response:**

**See comments above (response lines 131-138). This analysis has been done and is added to the paper. There is no substantive difference between N S and overall for hotspot analysis so it is not discussed here.**

*Reviewer:*

*L417 – 419 ‘…suggesting that they served as an effective control.’*

*I initially understood this to mean effective control for GW, rather than biases in the sampling of infections. I would suggest rewording this sentence to make it clear that you are referring to sampling bias.*

**Response:**

**Great point. This was indeed confusing. The sentence has been reworded to read: *“Importantly, these surveillance covariates accounted for a substantial amount of variation in parasite presence and hotspot identity, suggesting that the remaining variation between villages may be largely independent of differences in surveillance intensity.”* (ll. 451-454 in tracked changes document)**

Reviewer #3:

*Reviewer:*

*In relation to the take-home messages in the Abstract, please see my comments above and below.*

*More specifically, given that health-care worker visitation was a key factor in detection of infections, I would not consider this an "intrinsic" (or demographic, as in the previous paragraph) characteristic of the village.*

**Response:**

**This is a good point and we have clarified this and separated it from reference to demographic covariates throughout the paper.**

*Reviewer:*

*Climate variables are somewhat helpful as is elevation, but human population size and health worker visitation rate are the main contributors to the models' explanatory power. I think that when it comes to the models being "highly predictive" a little more care should be taken to relate the predictability to which factors are epidemiologically informative. There remain some assertive phrases, e.g. line 320-1, about how this might be used in targeting control efforts that I am not sure are strongly supported.*

**Response:**

**We have attempted to further soften our assertions throughout as we agree that the correlative findings are not necessarily identifying “drivers” per se and many of the important variables (e.g. dog population, number of healthcare worker visits) are not helpful for control. We would also note that informative parameters including fishing village identity and variation in elevation make up nearly as much explanatory power as human population size and health worker visitation (more in northern villages).**

--------------------

**Editorial and Data Presentation Modifications?**

Reviewer #1: (No Response)

Reviewer #3:

*Reviewer:*

*Thanks to the authors for having dealt with, or acknowledged and responded positively too, many of the substantive concerns in my initial review.*

*Nevertheless, I find I have a large number of presentational and editorial points. I think the paper would still be considerably better if these were addressed. I have tried not to be too particular, but some of these are quite important points. These primarily relate to the points raised above about being a bit more conservative in relation to the scope and outcomes of the study.*

*Title*

*Drivers: Given all the caveats that are now more thoroughly explored, it is probably not justified to use "Drivers" in the title. It might be better to use "Correlates of" or "Patterns in"*

*Infection in dogs: The analyses would better be a description of "villages in which dog infections are recorded" or some more accurate way of conveying the subject of the analysis, which is not of dog infections, but of recording detection of infections at a village level over an extended period.*

**Response:**

**We have reworded the title to better reflect this comment and our analysis the new title is:**

***Identifying correlates of Guinea worm (Dracunculus medinensis) infection in domestic dog populations***

*Reviewer:*

*Abstract*

*Guinea in Guinea worm is usually capitalised, as it derives from the country. (e.g. Line 38).*

**Response:**

**Thank you; we have corrected this to ensure Guinea is capitalized throughout the paper.**

*Reviewer:*

*Overall, I found the wording of the first Background paragraph of the Abstract to be problematic and imprecise. For example: It is not necessarily the case that the epidemiology changed, or that this happened "at the same time". Why is 2012 selected? Domestic dogs were observed to be hosts to Guinea worm multiple times and in multiple countries before 2012 and outside of Chad, and before they were found in Chad. It is not the dogs that challenge efforts exactly, they are indifferent, but the presence of infection in a non-human host creates a challenge for those charged with eradication. See my point above about "driving" and drivers. Although these would be good to know, this study does not, in the end, reveal them. I am sorry to be picky about these finer points, but attention to this sort of detail will help with the tone of the rest of the paper.*

**Response:**

**We have rewritten this section as we agree it is a bit imprecise/confusing (ll. 26-34 in tracked changes document). We would note that it is our understanding that the first confirmed cases of GW in dogs in Chad occurred in 2012 (Eberhard et al. 2014) though presumably they were present before this and we have tried to clarify this point. Thank you in particular for the *“presence of the parasite in a non-human host”* language.**

*Reviewer:*

*Might it be better throughout to talk about the "detection of infection" rather than "infection" per se? (e.g. Line 47)*

**Response:**

**While we change this here for the sake of clarity (l. 37 and l. 47 in tracked changes document), we do not change throughout as this is cumbersome and the distinction is true of all studies of parasites. It is implicit that data on “infection” includes a detection component.**

*Reviewer:*

*Similarly, it is not "Guinea worm parasite presence" but "detection of Guinea worm infection in a village" (Line 54).*

**Response:**

**As above this is changed here for clarity but not throughout the paper.**

*Reviewer:*

*I would suggest that n healthcare visitors is not "intrinsic to the village", since it is increased or decreased by the eradication program. (Line 54)*

**Response:**

**We have a removed this description here and throughout the paper**

*Reviewer:*

*I would also suggest that in terms of "landscape scale ecology" the insight is limited, since the relevant factors were not especially ecological. But this is a subjective point. (Line 56)*

**Response:**

**We changed this from ecology to epidemiology.**

--------------------

**Summary and General Comments**

Reviewer #1:

*Reviewer:*

*The authors have made appropriate responses to comments on the previous version of this manuscript, and many of the issues and concerns have been addressed. Overall, this is a well written manuscript and this body of work has the potential to significantly contribute to our understanding of a zoonotic disease that is on the brink of eradication. However, the methodology and results newly presented by the authors still raise considerable questions, and there are several areas for clarification/improvements that need to be addressed before the manuscript can be accepted for publication:*

*(1)	I have several concerns about the hotspot analysis. While figure 1 is much improved and helps the reader see the results of the spatial scan analysis, it is surprising to see that there is considerable overlap of hotspots. This suggests to me that there is an error as I would only expect overlaps if the data were temporal in nature. This either needs rectifying or requires some explanation in the methods.*

**Response:**

**This comment is discussed in detail above (response lines 198-212) but overlapping hotspots are to be expected with this analysis even without a time component. It simply selects all circular areas of a set of radii which have significantly more infections than expected based on dog population. This could occur in multiple areas that happen to overlap. Although other spatial scan statistics exclude overlapping areas, this particular statistic does not. We added text to clarify this in the methods and added an additional reference for those interested in delving deeper:**

***“Because the spatial scan is performed at varying radiuses centered on all villages, overlapping or concentric clusters are possible”* (ll. 205-206 in tracked changes document)**

*Reviewer:*

*(2)	The authors have appropriately adapted their analysis to consider collinearity however, there are some discrepancies that could require a re-run of the analysis. ‘Co-linear clusters were reduced to the single most central variable’, implying that the non-central variables were removed from the model, but this does not seem to be the case. This needs clarification as to if a different method was used or, alternatively, the models will need to be corrected and run again.*

**Response:**

**As referenced above (response lines 75-81) this was, in fact, an issue with table S3 rather than an issue with analyses. The only variables included in models were those not in a cluster nor the central variable in clusters. Note also that the present climate analysis had a different set of clusters.**

*Reviewer:*

*(3)	Interactions are included in models, but they are overlooked in the results and discussion. The interactions between variables will be important for the interpretation of the results and should be given more attention. Currently it is not clear why they are included in the model or how they should be interpreted.*

**Response:**

**This is discussed more fully above (response lines 93-123) but we include interactions because they are associated with a tuning parameter in our model. We include the supplementary tables and supplementary visualizations of the strongest interaction for the interested reader (and now reference them in the methods). But we removed any mention in results at suggestion of prior reviewers due to the lack of interpretability/explanatory power. This clarification is now made in the methods that the interactions were fit as an incidence of one of our tuning parameters and as interactions were not central to our analysis and proved uninformative they are excluded from the main text’s results and discussion.**

Reviewer #3:

*Reviewer:*

*As indicated above, I feel that although the response document acknowledge much of the earlier critique, the revisions have not sufficiently addressed the issue of scope and ambition of the analyses. In essence, this is an analysis of correlates of the village-level detection of infection over an extended period. It is not an analysis of the drivers of dog infections. Some of this change of tone is in the main text, but the Title, Abstract and Author Summary remain too assertive.*

**Response:**

**We appreciate the reviewers’ comments on changes made to the manuscript. We have made additional edits throughout to clarify the scope and interpretation of this analysis and do our best not to overstep. We have also (as discussed above) made edits to the title, abstract, and author summary to explicitly address specific comments relating to the tone of the article.**
