# Supplementary material for: Identifying correlates of Guinea worm (Dracunculus medinensis) infection in domestic dog populations
Source: PLoS Negl Trop Dis. 2020 Sep 14;14(9):e0008620. doi: 10.1371/journal.pntd.0008620 (PMC7515199; doi:10.1371/journal.pntd.0008620)
Supplement: Attachment — Submitted filename: GW BRT Third Response to reviewers.docx [file pntd.0008620.s021.docx]

Request was made for data publication.

**Response:**

**We were able to negotiate publication of a semi-anonymized aggregated format of the data used in this article. We have added 4 data files to the supplement and altered the supplemental code to incorporate this format of the data and replicate our analyses.**

<b>Editorial and Data Presentation Modifications?</b></br><br/>
Use  this section for editorial suggestions as well as relatively minor modifications of existing data that would enhance clarity. If the only modifications needed are minor and/or editorial, you may wish to recommend “Minor Revision” or “Accept”.

Reviewer #1: (No Response)

Reviewer #3: For the avoidance of doubt, none of these points is substantive and all are easily dealt with, with no further recourse to the reviewers.
L77 - This suggests that eradication might have been achieved in the 1980s, whereas the target of eradication was set in the 1980s.

**Response:**

**Revised sentence to read:**

**“With a historical distribution spanning 21 countries across Asia and Africa, in the 1980s, Guinea worm was initially targeted for eradication by the World Health Assembly” (ll. 76-78)**

L84 - I would say that it is not so much the annual occurrence as the persistence of human cases.

**Response:**

**Revised sentence to read:**

**“Since then, disease cases have persisted and all available evidence links the persistence of human cases to the emergence of Guinea worm in domestic dogs” (ll. 83-85)**

L85 - Should you add the species name for dogs?

**Response:**

**Added (*Canis lupus familiaris*) (ll. 85)**

L91-3 - Recent work has hypothesised this pathway, and identified some contributing elements, without demonstrating it.

**Response**

**While we agree with this statement we feel that the sentence as written does not overstate the evidence for the described mechanism (we do not use the language “demonstrate”).**

L138-9 and L259-50 - Clarify village-level prevalence, as this could still be misconstrued as the prevalence among dogs.

**Response:**

**Revised to (ll. 138-140, 150-152) :**

**“Over this five-year study period, the village-level prevalence (proportion of villages with an infection) of *D. medinensis* in dogs was 19.1% (406/2125), but the number of infections per village varied widely (e.g. 2016 range: 0-71), and generally increased over time.”**

**Left as is (ll. 259-260)**

**We feel that the percentages are fairly clearly explained at 259-260 and “prevalence” is not referenced.**

L139 and L250 - Clarify, this is dog infections and whether this is separate infections, or dogs with worms, as many dogs have >1 worm infection.

**Response:**

**The point is well taken we have revised to:**

**“Infections are only recorded when a *D. medinensis* worm emerges from the skin, becoming visible to Guinea Worm Eradication Program (GWEP) staff, thus an infection report reflects the definitive occurrence of a *D. medinensis* infection by a single worm.” (ll. 136-139)**

L322 - "Canine Guinea worm" implies that it is a dog parasite, better to say Guinea worm infection in dogs

**Response:**

**Revised to:**

**“We found that a combination of demographic, geographic, climatic, and surveillance variables were important predictors of detection of Guinea worm infection in dogs in the villages of Chad.” (ll. 323-324)**

L357 - It is not higher risk of Guinea worm infection in dogs, but greater likelihood of detecting dog infections in villages.

**Response:**

**The lack of the village level to this statement has been fixed. We have revised to:**

**“Our parallel finding that a fishing culture is associated with higher risk of Guinea worm infection in the dogs of a village suggests that addressing human behaviors that facilitate transmission through fishing-related activities may enhance disease control.” (ll. 358-361)**

L439 - Clarify that there is no transmission between dogs and humans, as such.

**Response:**

**We describe the transmission pattern above but add the word “indirect” to clarify that dogs and humans are not directly infecting each other. Revised to:**

**“This includes efforts already underway to elucidate new pathways involved in the parasite life cycle (e.g.** **(4,8,9,35)****) and to clarify the modes of indirect transmission between dogs and humans (e.g. (6)).” (ll. 440-442)**

--------------------

<b>Summary and General Comments</b></br></br>
Use  this section to provide overall comments, discuss strengths/weaknesses of the study, novelty, significance, general execution and scholarship. You may also include additional comments for the author, including concerns about dual publication, research ethics, or publication ethics. If requesting major revision, please articulate the new experiments that are needed.

Reviewer #1: The authors have made appropriate changes to the manuscript and provided clarification on the methods used. I have no further suggestions or queries and think this manuscript is acceptable for publication. I would like to congratulate the authors on producing a well written manuscript with interesting results that will help guide future research towards the control of Guinea worm disease.

**Response:**

**We thank the reviewer for their helpful feedback throughout this process!**

Reviewer #3: The authors have tackled my last set of suggestions and recommendations in good part,

Some minor points that are definitely optional but which might improve understanding and clarity are in the box above. None of these requires any further review or assessment.

**Response:**

**We thank the reviewer for their constructive feedback throughout this process. We make edits to address the minor points above.**

One easy point that does require correction, at L326, and which was raised previously is that it is not elevation that was found to be influential, but standard deviation in elevation.

**Response:**

**We have fixed this omission. Revised to:**

**“The size of the dog population and the status of a village as a fishing village were demographic predictors of Guinea worm infection at the village scale, while standard deviation in elevation emerged as a geographic predictor at this scale.” (ll. 326-329)**

I have no other concerns.
